# Use of Machine Learning for the Estimation of Down‐ and Up‐Link Field Exposure in Multi‐Source Indoor WiFi Scenarios

**DOI:** 10.1002/bem.22361

**Published:** 2021-07-23

**Authors:** Gabriella Tognola, David Plets, Emma Chiaramello, Silvia Gallucci, Marta Bonato, Serena Fiocchi, Marta Parazzini, Luc Martens, Wout Joseph, Paolo Ravazzani

**Affiliations:** ^1^ National Research Council, Institute of Electronics Computer and Telecommunication Engineering (CNR IEIIT) Milan Italy; ^2^ Department of Information Technology Gent University/IMEC Gent Belgium; ^3^ Department of Electronics, Information and Bioengineering (DEIB) Politecnico di Milano Milan Italy

**Keywords:** exposure assessment, indoor RF exposure, Machine Learning, Neural Network

## Abstract

A novel Machine Learning (ML) method based on Neural Networks (NN) is proposed to assess radio‐frequency (RF) exposure generated by WiFi sources in indoor scenarios. The aim was to build an NN capable of addressing the complexity and variability of real‐life exposure setups, including the effects of not only down‐link transmission access points (APs) but also up‐link transmission by different sources (e.g. laptop, printers, tablets, and smartphones). The NN was fed with easy to be found data, such as the position and type of WiFi sources (APs, clients, and other users) and the position and material characteristics (e.g. penetration loss) of walls. The NN model was assessed using an additional new layout, distinct from that one used to build and optimize the NN coefficients. The NN model achieved a remarkable field prediction accuracy across exposure conditions in both layouts, with a median prediction error of −0.4 to 0.6 dB and a root mean square error of 2.5−5.1 dB, compared with the target electric field estimated by a deterministic indoor network planner. The proposed approach performs well for the different layouts and is thus generally used to assess RF exposure in indoor scenarios. © 2021 The Authors. *Bioelectromagnetics* published by Wiley Periodicals LLC on behalf of Bioelectromagnetics Society.

## INTRODUCTION

The use of Machine Learning (ML) to solve electromagnetic problems is a recent topic. Typical applications are quickly arising, for example from the 5 G world, where ML is applied in smart antenna design (for a review, see Rawat et al. [2012]). In the present study, we propose an ML method based on Neural Networks (NN) for the estimation of field exposure generated by multiple WiFi sources (2,400 MHz) in an indoor scenario. Assessment of indoor radio‐frequency (RF) exposure is indeed an important and timely issue considering the ever‐increasing number of RF devices being used in everyday life, including also connected objects in the Internet of Things (IoT) [Varsier et al., 2015; Chiaramello et al., 2019a]. The estimation of field exposure in such a scenario is not trivial due to the complexity and variability of the setup, which should take into account the effects of multiple and diverse sources and the variability of the position of the sources in the room. Such exposure scenario cannot be modeled with deterministic methods only but requires the application of novel advanced statistical approaches (such as stochastic dosimetry and ML) capable of modeling the complexity and variability of the setup. For example, a few recent studies demonstrated that stochastic dosimetry could be valuable for assessing how the variability of sources' position in the room affects the dose of exposure in indoor setups [Chiaramello et al., 2018, 2019b,c].

One common way to derive the dose of exposure in indoor (or outdoor) contexts is first to estimate the field exposure, or more generally the wave propagation [Plets et al., 2015; Varsier et al., 2015]. In the past, wave propagation and the corresponding path loss models typically applied deterministic methods based, e.g. on ray‐tracing [Ji et al., 2001] or heuristic algorithms [Plets et al., 2012]. On the one hand, deterministic propagation models can provide accurate estimations of path loss. However, on the other hand, they require time‐consuming and heavy computations that must be entirely run from scratch once the propagation environment has changed. Vice versa, propagation models based on ML are computationally very efficient and can be used to solve new environments or propagation conditions different from those used to initially train the model, thus being extremely attractive in real‐life applications.

The application of ML to solve path loss problems in indoor (residential or office buildings) and outdoor (urban settings) scenarios is a recent topic. For indoor scenarios [Neskovic et al., 2000; Zineb and Ayadi, 2016; Trogh et al., 2019], the input variables to the NNs were the relative distance from the emitting antenna(s) and other elements that characterize the settings, such as the position and material of walls, corridors, doors, windows, etc. For outdoor scenarios [Piacentini and Rinaldi, 2011; Zhang et al., 2018, 2019; Popoola et al., 2019; Jo et al., 2020], the input variables were typically the frequency and the height of the transmitting and the receiving antennas, the buildings' height, and the distance between the base station and the mobile station. As illustrated in all these latter studies, the estimation of path loss with NNs was very accurate, if not even better than that achieved with deterministic approaches.

To the best of the authors' knowledge, all past studies that applied ML for path loss estimation addressed only the case of wave propagation under down‐link (DL) transmission by one or multiple antennas that serve as APs (in indoor scenarios) or base stations (in outdoor scenarios). The novelty of our approach consists in the realization of a new and generalized NN model capable of addressing for the first time the inherent variability and complexity of realistic indoor scenarios, including both the contribution of DL transmission by APs and up‐link (UL) transmission by a great variety of sources (e.g. laptop, printers, tablets, and smartphones). To account for both DL and UL exposure, we defined a totally different architecture of the NN model: as a matter of fact, in addition to input variables related to only APs (as already done in past studies), we defined a novel and different NN model that also takes into account a set of input variables specifically related to UL (e.g. number, position, and characteristics of WiFi sources). Such input variables were never used in past approaches. This is thus the first study where a novel NN architecture based both on DL and UL input variables is tested and assessed. The model is here assessed for WiFi sources at 2,400 MHz, which are an important contribution to indoor exposure in our daily lives [Varsier et al., 2015].

The aim of the present study was to develop and assess a tool—a NN model—capable of predicting the exposure field in general indoor settings, different from the one used to build and train the NN model. The goal is to predict the exposure field in a new layout by simply feeding the proposed NN model with the number and positions of sources, the geometry of the building/room, and penetration losses of the walls of the new layout, without building from scratch a new NN model. In the present paper, we describe the rationale and the procedure we used to build and optimize the NN model. We also provide an extensive quantitative assessment of the prediction accuracy of the proposed NN model on a layout different from that one used to train the NN, showing the applicability of the proposed approach.

## METHODS

The proposed method is a generalization of available ML approaches and has the advantage to be more suitable to address real‐life applications. In our approach, an NN is fed with easy to be found data, such as the position and type of WiFi sources (APs and clients/users such as laptops, printers, tablets, and smartphones) and the position and material characteristics (e.g. penetration loss) of walls. To assess the accuracy of our NN, we compared the electric field estimated by the NN with the electric field calculated in indoor scenarios by a state‐of‐the‐art deterministic approach [Plets et al., 2012]. We used the deterministic approach of [Plets et al., 2012] as a tool to derive a reliable picture of the distribution of the field generated by WiFi sources; the electric field calculated with the method of [Plets et al., 2012] was thus considered by us as the true electric field that our NN has to estimate. Finally, we compared the prediction accuracy of our NN model with that achieved with similar approaches that used ML for electric field estimation in indoor layouts [Zineb and Ayadi, 2016; Neskovic et al., 2000; Trogh et al., 2019].

### The Simulated Indoor Layouts

**Figure 1:**
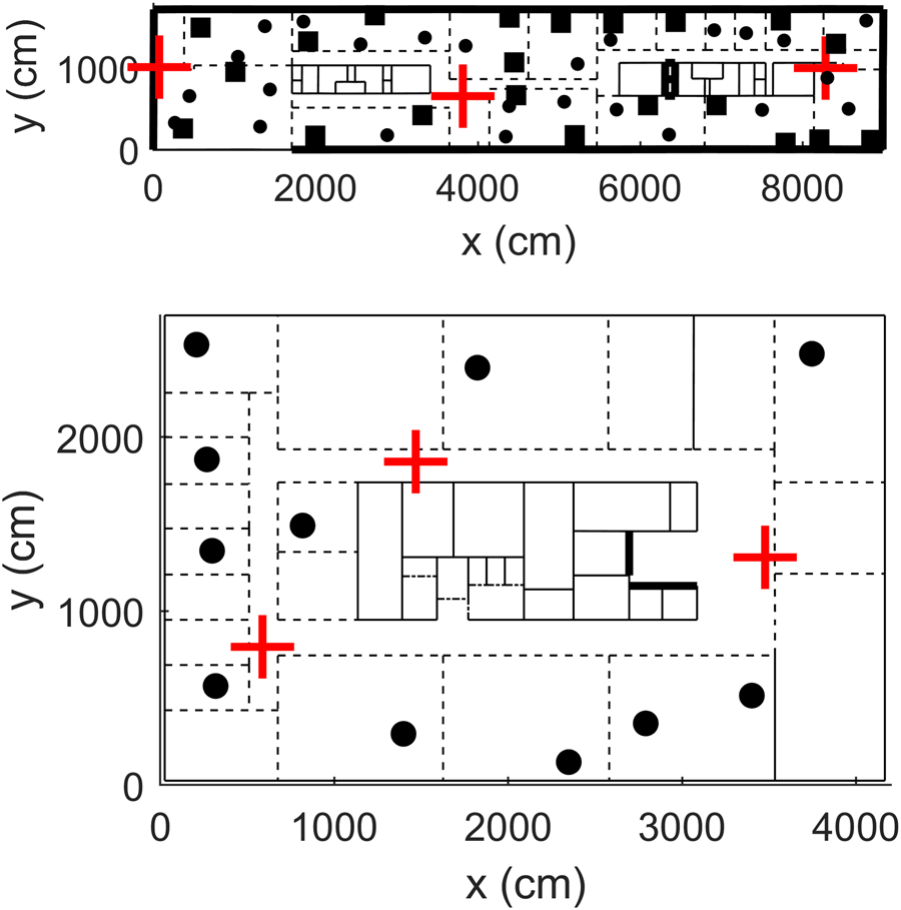
The simulated layouts used to build and optimize the NN model (top panel) and to assess the NN accuracy (bottom panel). The two layouts are office buildings (top layout: 90 × 17 m^2^; bottom layout: 42  × 27 m^2^) with walls made by concrete (thin solid lines, 10 dB penetration loss), layered drywall/glass (dashed lines, 2 dB penetration loss), wood (dash‐dot lines, 6 dB), and metal (thick lines, 100 dB penetration loss). Wall thickness was 10 cm. Inside the buildings, there are WiFi APs (cross symbols), WiFi clients, such as printers, laptops, and TVs (squares), and other WiFi users, such as smartphone/tablet users (circles). AP = access point; NN = Neural Network.

We considered two different indoor layouts whose characteristics resemble those of typical residential setups. The two indoor layouts are realistic in the sense that they include walls at different positions and of different materials and RF sources at different positions, transmitting in DL and in UL. Both layouts are two‐dimensional environments and did not consider the presence of the furniture and other large equipment. On top of Figure 1 is the layout we used to build and optimize the NN model and to perform a preliminary assessment of the NN accuracy; at the bottom of Figure 1 is the layout we used to perform a deeper assessment of the previously determined NN model. We placed inside the two layouts walls of different materials and different penetration losses. In both layouts, we simulated three WiFi APs at 250 cm above the ground (with maximal Equivalent Isotropically Radiated Power (EIRP) of 20 dBm, operating in the 2.437 GHz band), WiFi‐only clients (e.g. printers, TV, and laptops), and WiFi users (e.g. smartphones and tablets), placed at a typical working height of 130 cm above the ground level. WiFi UL power was set at 20 dBm as well, with a duty cycle of 2% [Joseph et al., 2013].

### The True Exposure Field

We used the WHIPP algorithm [Plets et al., 2012, 2013a,b, 2015], a heuristic planning algorithm developed and validated for the prediction of path loss in indoor environments, to derive the exposure field *E* (*V*/*m*) generated by the WiFi sources inside the two layouts of Figure 1. This field was considered as the “true” value, while the field derived with the NN was considered as the “predicted” value. In such a multi‐source scenario, the exposure field is not always the one generated by the dominant source, i.e. the source closest to the point of measurement. This can be seen, e.g. when a thick concrete wall is between the two points. Moreover, even when the strongest contribution is determined, the total field can still significantly deviate when multiple sources cause a similar field value at the considered location. Per AP or client, the electric field strength is calculated by WHIPP based on a realistic duty cycle [Joseph et al., 2013]. The field values are then combined as follows [Plets et al., 2013a]:

(1) $$E_{\mathrm{total}}=\sqrt{\overset{N}{\underset{i=1}{\sum }}E_{i}^{2}}$$

where *N* is the number of transmitting sources on the building floor, *E*_*i*_ is the electric field strength caused by the transmitting source *i*. The field vectors caused by the different sources are thus assumed to have no phase correlation.

As described in the section Characteristics of the Neural Network Models Across Exposure Conditions, in our layouts we did not consider furniture and clutter. Ray‐tracing tools do account for furniture and clutter in general, but it was found that modeling these is very sensitive to the exact location and size of the clutter, and moreover, that there is a very large variability in estimated electromotive force levels, depending on the settings of the ray‐tracer [Plets et al., 2012]. Therefore, in the present study, we followed the heuristic approach WHIPP, based on the electromagnetic propagation physics, whereby we account for the dependence on distance, material losses, and diffraction around corners. WHIPP takes into account the more larger‐scale building characteristics and was shown in an experimental validation campaign to better reflect real values [Plets et al., 2012].

We calculated the true exposure field with WHIPP in five different exposure conditions: the first three were calculated using the layout at the top panel of Figure 1 and were used to build and optimize the NN models; the last two exposure conditions were calculated using the new layout shown at the bottom panel of Figure 1 and were used to assess the NN accuracy. Thus, to build the NN model, we considered three exposure conditions, namely: (i) down‐link exposure due to APs only (DL); (ii) down‐link + up‐link exposure due to APs and WiFi clients (DL + UL | clients); and (iii) down‐link + up‐link exposure due to APs, WiFi clients, and other users, such as tablets and smartphones (DL + UL | clients&users). Finally, to assess the NN accuracy, we considered two exposure conditions, namely: (iv) down‐link exposure due to APs only (DL | new); and (v) down‐link + up‐link exposure due to APs and WiFi clients (DL + UL | new). For all conditions, the electric field was calculated at a height of 130 cm above the ground on a 20‐cm regular grid spanning the entire buildings. This resulted in a dataset of a total of 38,500 samples of E for DL, DL + UL | clients, and DL + UL | clients&users conditions and 27,400 samples of E for DL | new and DL + UL | new conditions.

### NN Models' Implementation

We implemented three feed‐forward NNs [Dreyfus, 2005] to model the exposure field in DL, DL + UL | clients, and DL + UL | clients&users conditions, respectively. The NN was implemented using the Deep Learning Toolbox of Matlab (Matworks, Natick, MA). A feed‐forward NN is a multilayer network of interconnected artificial neurons. The NN is used to estimate the output (i.e. the electric field E inside the buildings) after it has been fed with the inputs (i.e. the independent variables). To do this, the NN is trained to learn which is the output that corresponds to given inputs, using a set of examples called “training set” for which the inputs and the corresponding true outputs are known. During training, the coefficients of the NN model are iteratively adjusted to minimize the difference between the output predicted by the network and the true known output. In the present study, we used the Levenberg‐Marquardt backpropagation method [Levenberg, 1944; Marquardt, 1963] to iteratively adjust the NN coefficients to minimize the Training Mean Square Error *TMSE* between the predicted and the true output of the observations in the training set:

(2) $$TMSE=\frac{1}{N_{T}}\overset{N_{T}}{\underset{k=1}{\sum }}{\left[{{{y_{pred}}_{k}-y}_{true}}_{k}\right]}^{2}$$

where *N*_*T*_ is the number of observations in the training set, $y_{true_{k}}$ is the true output of observation *k* and $y_{pred_{k}}=g(x_{k},w)$ is the output predicted by the NN by using the vector of inputs ***x***_*k*_ and the vector of network coefficients ***w***, for observation *k*.

To determine the optimal number of layers to prevent NN overfitting to the training data, we applied the cross‐validation technique [Stone, 1974]. This technique aims to estimate the Cross‐Validation Score *CVS*; that is, the error the NN would make in predicting the output from inputs never used, as a function of the number *i* of layers. In this study, we considered NNs with a number *i* of layers increasing from 1 to 10. Cross‐validation involves partitioning the training dataset in *K* disjoints subsets while using *K*−1 subsets to adjust the network coefficients (by minimizing *TMSE*) and the remaining *K*‐th subset to calculate the *K*‐th Mean Square Error *KMSE*, which is the error between the predicted and the true output on the samples of the *K*‐th subset. The procedure is iterated *K* times until each subset of the *K* subsets has been used. In our study, we partitioned the data into *K* = 10 subsets. The *CVS*(*i*) for a network of i=1,⋯,10 layers was calculated as [Stone, 1974]:

(3) $$\mathrm{CVS}(i)=\frac{1}{K}\overset{K}{\underset{j=1}{\sum }}{\mathrm{KMSE}}_{j,i}$$

where *KMSE*_*j,i*_ is the *KMSE* of partition *j* obtained with an NN with *i* layers. The procedure is terminated when *CVS*(*i*) calculated for a given number *i* of layers started increasing, meaning that overfitting occurred. The optimal number of layers was then determined as the number of layers obtained immediately before overfitting occurred, i.e. at *i*−1.

### Input Variables of the NN Models

As illustrated in the Introduction, we developed a novel NN architecture where we defined input variables to account for DL and UL exposure effects separately. For DL exposure conditions, the NN model was fed with the number of APs, the number and the average penetration loss of non‐metallic walls, and the number of metallic walls near the point in the building where the electric field has to be estimated. For the NN that modeled the DL + UL | clients condition, in addition to the inputs considered in the DL condition we defined an additional set of input variables—the number and position of WiFi clients (i.e. laptops, printers, and TVs)—to specifically account for the exposure field generated during UL. Finally, for the NN that modeled the DL + UL | clients&users condition, we considered all the inputs previously listed and additional variables that accounted for the number and position of WiFi users (e.g. tablets and smartphones). All variables above were calculated at distances from 50 cm to 5 m with a step of 50 cm from the observation point, i.e. the point in the building where the electric field has to be estimated. Roughly speaking, we passed to the NN model the information relating to the geometry of the building and to the walls' and sources' position not as absolute coordinates but in a relative way, i.e. relative to the observation point. In this way, the NN model can predict the correct exposure field in new layouts never seen during training because it has been built not on the “absolute” positions of walls and WiFi sources, but on the positions relative to the observation point.

The total number of input variables for the NN models of DL, DL + UL | clients, and DL + UL | clients&users conditions was 40, 50, and 60, respectively. A detailed description of the input vector is included in the Supplementary Appendix.

### NN Training

The three NN models were trained on the exposure field calculated by WHIPP using the layout on top of Figure 1. We divided the available samples (*N* = 38,500 points) into two disjoint subsets, namely in the *training* (*N* = 30,800) and *test* set (*N* = 7,700), where the training set was used to build and optimize the NN coefficients and the test set was used to preliminarily assess the performance of the NN on data from the same layout that was not used during training. To maximize the variability within the training set and between the training and test sets, we subdivided the points on the 20‐cm grid spanning the entire layout in blocks of 100 points each and put 80% of the blocks in the training set and the remaining 20% in the test set.

### Assessment of NN Prediction Accuracy

We performed first a preliminary assessment of the NN accuracy by using the test set (*N* = 7,700 points) from the layout used to build the NN model (i.e. the layout on top of Fig. 1) and then a deeper assessment by using a different and a new layout (*N* = 27,400 points), shown at the bottom of Figure 1. For both layouts, we calculated the correlation value *R* to measure how the true and the predicted output were linearly related:

(4) $$R=\frac{s_{E_{\mathrm{true}},E_{\mathrm{pred}}}}{s_{E_{\mathrm{true}}}s_{E_{\mathrm{pred}}}}$$

where

$$s_{E_{\mathrm{true}},E_{\mathrm{pred}}}=\frac{1}{N-1}\overset{N}{\underset{k=1}{\sum }}\left({E_{\mathrm{true}}}_{k}-\bar{E_{\mathrm{true}}})({E_{\mathrm{pred}}}_{k}-\bar{E_{\mathrm{pred}}}\right)$$

is the sample covariance, $s_{E_{\mathrm{true}}},s_{E_{\mathrm{pred}}},\bar{E_{\mathrm{true}}},\text{and}\bar{E_{\mathrm{pred}}}$ are the sample standard deviations and means calculated over the samples ${E_{\mathrm{true}}}_{k}$ and ${E_{\mathrm{pred}}}_{k}(k=1,\cdots ,N)$ of the true and predicted electric field and *N* is the number of samples. The higher *R*, the closer is the predicted output to the true output and the better is the fit of the NN.

We also evaluated the bias Δ*db* between the predicted and the true electric field and its root mean square error *RMS*_Δ*db*_ expressed on a dB scale, as also done by similar studies (e.g. Neskovic et al. [2000]; Zineb and Ayadi [2016]; Trogh et al. [2019]):

(5) $${∆\mathrm{db}}_{k}=20\log_{10}\frac{{E_{\mathrm{pred}}}_{k}}{{E_{\mathrm{true}}}_{k}}$$

(6) $${\mathrm{RMS}}_{∆\mathrm{db}}=\sqrt{\frac{1}{N}\overset{N}{\underset{k=1}{\sum }}{{∆\mathrm{db}}_{k}}^{2}}$$

## RESULTS

### Characteristics of the NN Models Across Exposure Conditions

**Figure 2:**
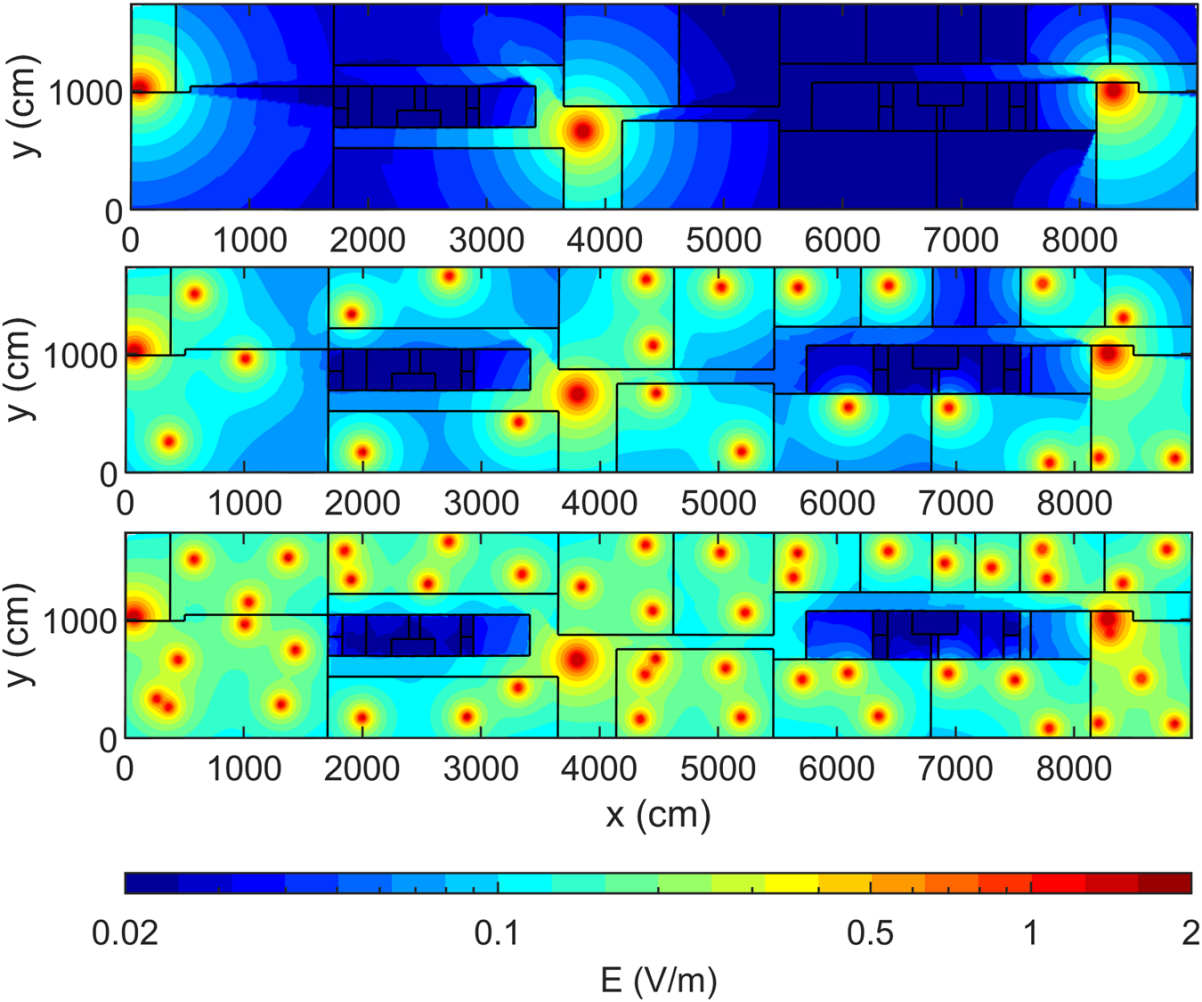
Distribution of the true electric field *E* for the exposure layout used to build and optimize the NN model (Fig. 1, top panel). The electric field was calculated by WHIPP for DL (first row), DL + UL | clients (second row), and DL + UL | clients&users exposure condition (third row).

Figure 2 shows the distribution of the true electric field that was used to build and train the three NN models for DL exposure (*E* median: 40 mV/m; first quartile Q1: 20 mV/m; third quartile Q3: 70 mV/m; standard deviation (SD): 114 mV/m), DL + UL | clients exposure (median: 120 mV/m; Q1: 80 mV/m; Q3: 180 mV/m; SD: 153 mV/m), and DL + UL | clients&users exposure (median: 190 mV/m; Q1: 140 mV/m; Q3: 260 mV/m; SD: 183 mV/m).

Table 1 shows the characteristics (number of layers, *MSE*, and *R*) of the three NN models. As seen in Table 1, the optimal number of layers was similar for the three models and equal to 5 for DL and DL + UL | clients&users conditions and 4 for the DL + UL | clients condition. This optimal number of layers was identified from the analysis of the values of *KMSE* as a function of the partition number and the number of layers and of the cross‐validation *CVS* function by varying the number of layers. A detailed description of *KMSE* and *CVS* values is included in the Supplementary Appendix. The *MSE* increased from DL to the DL + UL | clients&users exposure condition, thus indicating that the fit of the NN was better in the DL condition (lower *MSE*) than in the other two exposure conditions (higher *MSE*). Finally, Table 1 shows the correlation value *R* between the true and the predicted output for the training and test set. *R* was very high for all exposure conditions and ranged from 0.93 to 0.96, indicating that the fit of the NN to true output was very good. It is also seen that *R* was high even in the worst condition, i.e. for the test set, for which *R* was 0.93–0.94.

**Table 1:** Neural Network Characteristics Across Different Exposure Conditions

Quantity	DL	DL + UL | Clients	DL + UL | Clients&users
Number of layers	5	4	5
*MSE* (mV/m)^2^			
Training	9 × 10^−4^	23 × 10^−4^	38 × 10^−4^
Test	25 × 10^−4^	27 × 10^−4^	32 × 10^−4^
*R*			
Training	0.96	0.95	0.95
Test	0.94	0.93	0.93

MSE and *R* were calculated from the training (*N *= 30,800 points) and test set (*N* = 7,700 points) of the exposure layout displayed on top of Figure 1.

The computational time for building the NN models described in Table 1 and optimizing the model coefficients was about 30 min on a 2 core computer using Intel i7 CPUs @2.80 GHz.

### NN Accuracy

**Figure 3:**
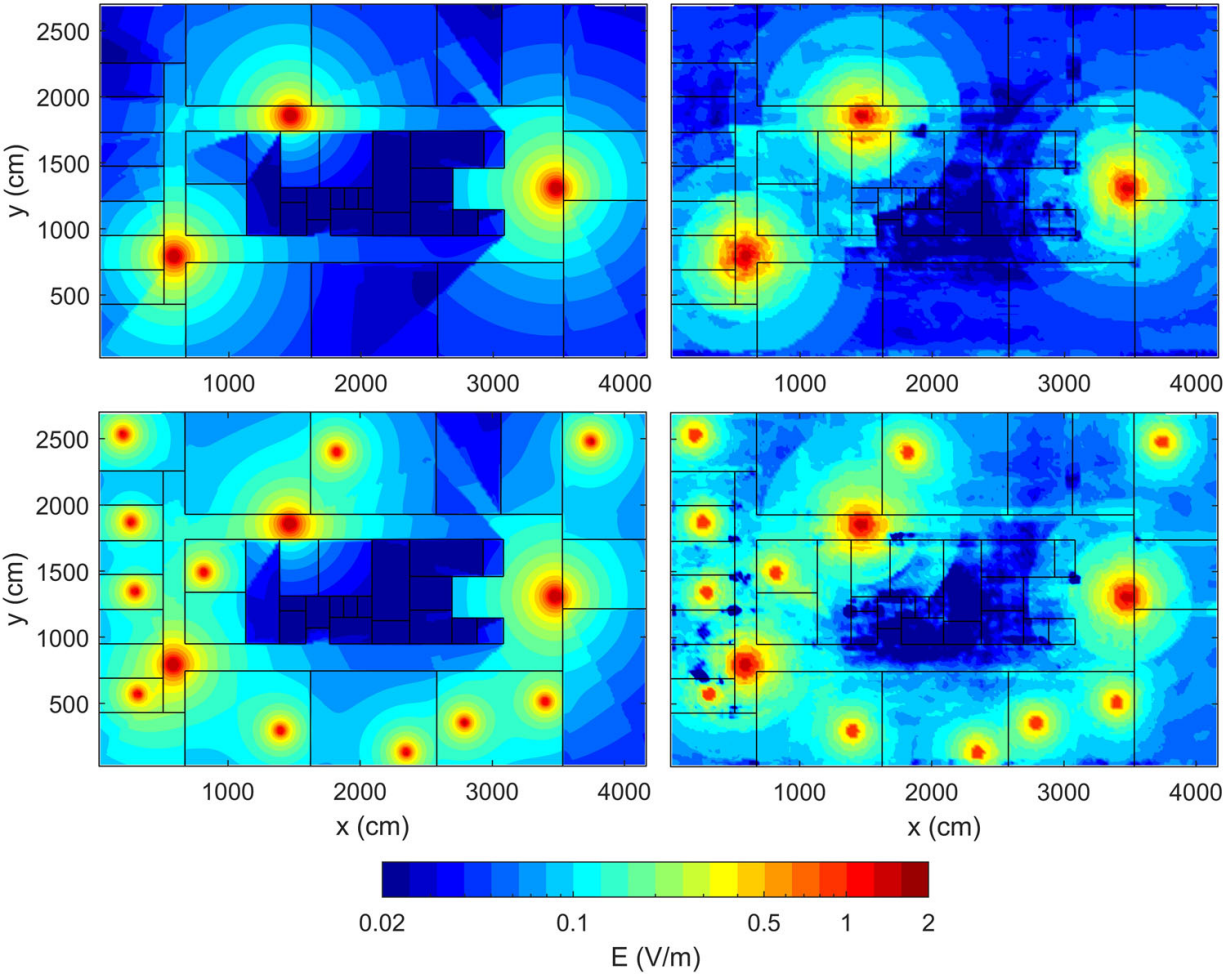
Distribution of the true (left column) and the predicted electric field (right column) for DL | new (first row) and DL + UL | new (second row) exposure conditions of the new layout shown in Figure 1, bottom panel.

Figure 3 shows the distribution of the true and the predicted exposure field in the DL | new and DL + UL | new conditions, that is the exposure conditions used to assess NN accuracy. The exposure field in DL | new and DL + UL | new conditions were predicted by using the NN models fitted on the DL and DL + UL | clients conditions, respectively (see section Characteristics of the Neural Network models across exposure conditions of the Results). The computational time to predict the electric field in the new layout by re‐using the NN models previously built and optimized was less than 1 min. From a qualitative point of view, it is possible to see at a first glance from Figure 3 that the field distribution calculated by the NN on this new layout, which is different from that one used to train the NN, was very similar to the true field distribution, for both exposure conditions.

**Figure 4:**
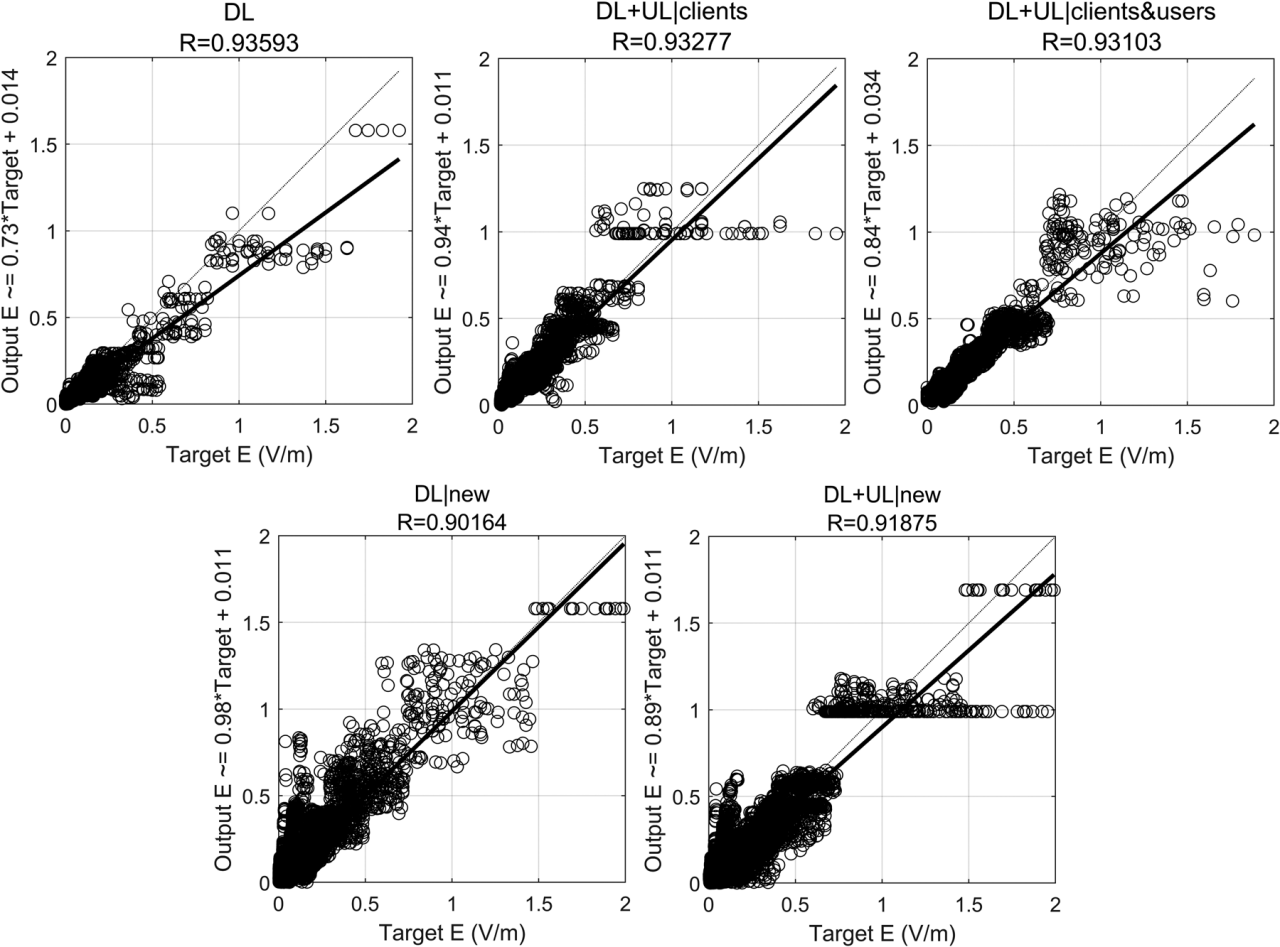
Linear regression fit between the electric field predicted by the NN model (*y*‐axis) and the true electric field (*x*‐axis) across exposure conditions. Top row: regression calculated on the test set (*N* = 7,700 points) of the layout used for NN implementation. Bottom row: regression calculated on the dataset of the new layout (*N* = 27,400 points). The straight bold line in each panel is the regression line, whose equation is displayed on the *y*‐axis. *R* is the correlation value between the target and the output.

To further investigate the accuracy, Figure 4 shows the linear regression between the true and the predicted electric field. For completeness, Figure 4 shows the regression calculated both on the samples of the test set of the layout used to build and optimize the NN (top row) and on the samples of the new layout (bottom row). It is observed that in all exposure conditions and both layouts, the output predicted by the NN models was very close to the true output, as indicated by the high *R* and the slope of the linear regression fits that is close to 1 (i.e. to the perfect match between true and predicted outputs). As expected, the NN fit was slightly lower for the new layout, when compared to the layout used to build and optimize the NN models. However, the fit for the new layout was good as demonstrated by the high *R*, ranging from 0.90 to 0.92.

Table 2 shows the bias Δ*db* and *RMSΔdb* across exposure conditions for the samples of the test set of the layout used for NN implementation and for the new layout. The bias Δ*db* between the predicted and the true electric field was small for both layouts: overall, the median value across layouts and exposure conditions ranged from −0.4 to 0.6 dB. As seen in Table 2, *RMSΔdb*, which is another measure of the prediction accuracy of the NN model, ranged from 2.5 to 4.8 dB for the test set of the layout used for NN implementation; for the new layout, *RMS*_*Δdb*_ was slightly higher and ranged from 4.6 to 5.1 dB. This result is somewhat expected and means that the prediction accuracy was slightly better when the NN is tested on samples coming from the same layout used for NN implementation (but in any case, different from the samples used during training) than when it is tested on a totally different layout. However, it is to note that even in the new layout, the NN accuracy was still good. Also, Table 2 shows that for both layouts the NN accuracy was slightly lower for the DL exposure condition for which both *Δdb* and *RMS*_*Δdb*_ were higher than those obtained in the DL + UL conditions.

**Table 2:** Neural Network Prediction Accuracy Across Exposure Conditions for the Test Set of the Layout Used for NN Implementation (*N* = 7,700 Points) and for the New Layout (*N* = 27,400 points)

		Layout used for NN implementation		New layout	
Quantity	DL	DL + UL | clients	DL + UL | clients&users	DL | new	DL + UL | new
*Δdb* (dB)					
Median	0.6	0.0	0.2	0.3	−0.4
Q1	−1.7	−1.0	−0.7	−1.4	−1.9
Q3	2.7	1.5	1.0	2.4	1.2
*RMSΔdb* (dB)	4.8	2.9	2.5	5.1	4.6

**Figure 5:**
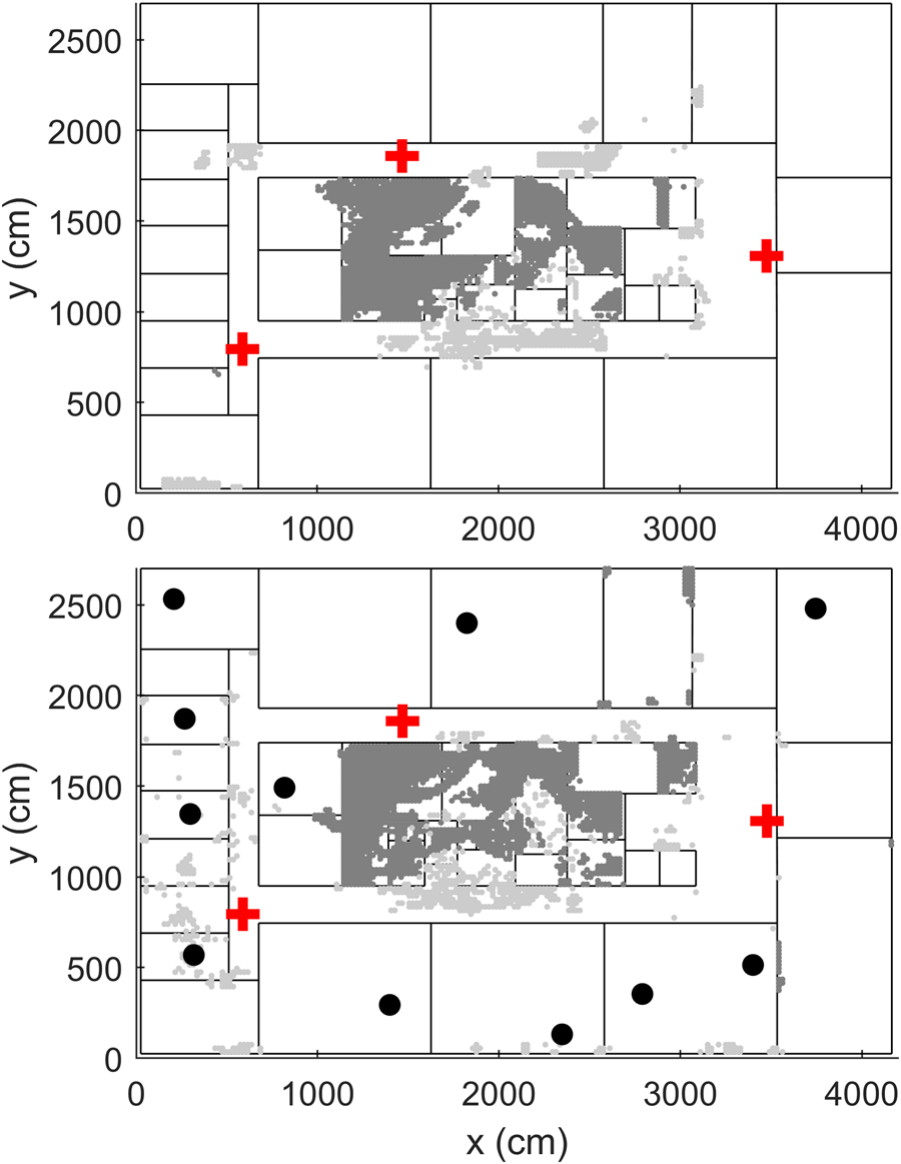
2D map showing the position inside the new layout of the outliers of *Δdb* for DL | new (top panel) and DL + UL | new (bottom panel) exposure conditions. The light gray points show the positions where *Δdb* was lower than Q1 − 1.5 × IQR (lower outlier value), while dark gray points show positions where *Δdb* was higher than Q3 + 1.5 × IQR (higher outlier value), with IQR = Q3–Q1. Red crosses are the APs; black circles are WiFi users. AP = access point; DL = down‐link; IQR = interquartile range; UL = up‐link.

Finally, Figure 5 shows the position inside the new layout of the outliers of *Δdb*, i.e. the positions inside the building where the NN model prediction was less accurate. For DL | new condition, the outliers were equal to *Δdb* < −7.1 dB and *Δdb* > 8.2 dB; for DL + UL | new condition, the outliers were *Δdb* < −6.5 dB and *Δdb* > 5.7 dB. For both exposure conditions, the outliers were located only at the boundaries of walls with higher penetration loss, i.e. the walls made by concrete (10 dB loss) and metal placed in the center of the building. In particular, inside the rooms surrounded by concrete or metal walls, the NN model tended to overestimate the exposure field; whereas immediately outside these rooms, the NN model predicts an exposure field lower than the true one.

## DISCUSSION

### General Performance of the NN

This paper presents the first results of the application of NN, an ML approach, to estimate the electric field generated indoors by multiple WiFi sources, considering the effect of both DL and UL transmissions by APs, WiFi clients (e.g. printers, TV, and laptop), and other WiFi users (e.g. tablets, smartphones, etc.). The NN was trained on a typical and realistic indoor exposure scenario whose characteristics and variability allowed building a generalized model of the indoor exposure field that would fit any specific environment or propagation condition similar to that investigated in the present study. The NN relied on a set of input variables that could be either obtained with affordable technology or from accessible technical datasheets. Namely, the input variables were the number and type of WiFi sources, the number of non‐metallic and metallic walls, and the average penetration loss of non‐metallic walls at distances increasing from 50 to 5 m from the point in the room where the electric field had to be estimated.

The NN accuracy was assessed on a new layout similar but distinct from that one used to build and optimize the NN model. The performance of the proposed approach was very promising: it was shown that once the NN model is trained, it can be safely re‐used to calculate the exposure field in different layouts. As a matter of fact, the accuracy for the new layout *Δdb* < 3 dB and the RMS error is similar for the first and new layouts, showing the applicability of the proposed approach. This aspect might be very important from a practical point of view, in particular regarding computational time. As reported in the previous sections, the time required to build and train the NN model was about 30 min. Compared to WHIPP, the time required could be around one hour for a building, but while WHIPP has to be recalculated every time the layout changes, the proposed NN model once trained can be re‐used in different layouts, taking only 1 min to calculate the new exposure field.

The performance of the NN in estimating the true electric field was very good as demonstrated by the high *R* that in the worst case, i.e. for the samples of the new layout, ranged from 0.90 to 0.92 across exposure conditions. Considering *Δdb*, which is a measure of the bias between the predicted and the true electric field on a dB scale, it resulted that the median value of the error *Δdb* calculated on all the positions of the new layout was low and equal to 0.3 dB for DL | new exposure and −0.4 dB for DL + UL | new exposure condition. This means that our approach could estimate the indoor electric field with a median error lower than the ±3 dB uncertainty typically obtained in experimental measurements.

The NN was found to overestimate the exposure field inside the areas surrounded by walls of high penetration loss (concrete or metal walls). This behavior might be due to a possible underestimation of the attenuation effect of the walls by the NN model. We are currently investigating this aspect; in particular, we are assessing the NN performance after introducing an additional set of input variables that will take into account not only the position of the walls (as already done in the present NN models) but also the number of walls along the straight path from the point in the room and the sources.

The present NN model was trained and validated on layouts whose top view had a rectangular shape, as it would be in many real indoor layouts. Nonetheless, it would be interesting to assess the performance of the current NN model with layouts with a top view less regular than a rectangle, i.e. with concave/convex layouts. It is interesting to note that the current model was already partially assessed in non line‐of‐sight (NLOS) propagation conditions that would occur with concave/convex layouts: as a matter of fact, inside the layouts already used in the current study, there were L‐shaped and T‐shaped corridors that deviated the field from direct propagation. As to WHIPP, the performance in estimating the path loss in concave/convex layouts is described in Plets et al. [2012].

### Comparison of NN Accuracy Across Different Exposure Conditions

*RMS*_*Δdb*_ was lower for both layouts in DL + UL conditions than in the DL condition. This might be a consequence of the normalization of *Δdb* to the magnitude of the electric field, which was greater in DL + UL than in DL | condition. Also, this might be due to the uncertainty of prediction increases with the distance from the sources.

### Comparison With Existing ML Literature Relating to Exposure Field Estimation

Although none of the past studies used the same approach as in our study, it is interesting to make a comparison, at least from a qualitative point of view with recent approaches using ML in the context of wave propagation in an indoor environment. As a general remark, the performance of our method was similar or even better than that of previous approaches based on ML that addressed exposure setups less complex than in our study. For example, Trogh et al. [2019] used ML to build and optimize radio maps for indoor positioning systems based on path loss estimation. In this study, an initial radio map computed by a theoretical path loss model is optimized with an unsupervised ML technique using unlabeled training data. The paper describes the application of the technique to optimize the radio map of an office building covering an area of 1,100 m^2^. The training data consisted of a random walk covering 900 different locations in the building. The test data for the validation are the 200 static locations in the same building. The average absolute error between the true path loss measured experimentally and the one predicted by Trogh et al. [2019] ranged from 2.9 to 4.0 dB, depending on the model used to initialize the radio map. In our work, the median value of *Δdb*, which is a measure similar to the path loss estimated in Trogh et al. [2019], was lower than in Trogh et al. [2019] and equal to 0.3 dB in DL | new exposure and −0.4 in DL + UL | new exposure, thus indicating that our approach gave comparable and even slightly better outcomes than in Trogh et al. [2019]. Differently from Trogh et al. [2019], we did not estimate the effect of human body shadowing on the accuracy of the exposure field predicted by our NN models. This is a very interesting aspect that we will consider in further releases of our NN model.

Neskovic et al. [2000] used NN to predict the electric field generated indoors by a single antenna operating in the 900 MHz band. Their NN was trained with the electric field measured at 297 positions inside the examined building. The accuracy of the NN was assessed using field values measured at additional locations in the building not used for NN training and also using values measured in a new building. Neskovic et al. [2000] considered as input variables the distance of the point in the building relative to the antenna and to other elements in the building, such as walls, corridors, doors, windows, etc. Differently from us, they built an NN model that accounted only for the presence of a single emitting antenna, whereas our NN model was capable of predicting the electric field produced in a far more complex scenario by multiple antennas both in DL (APs) and UL (clients and users). In Neskovic et al. [2000], the mean prediction error of the NN ranged from −5.3 dB to 2.4 dB across different setups of the building and of the position of the emitting antenna. These values were an order of magnitude higher than the median error obtained with the method proposed by us, which ranged from −0.4 to 0.6 dB across the tested layouts and exposure conditions.

Zineb and Ayadi [2016] proposed a model based on NN to predict path loss in an indoor environment (a University building) for frequencies in the 900, 1,800, 2,100, and 2,400 MHz bands. Their NN model was trained with real data measured in a building covering an area of 793 m^2^. The available dataset consisted of 260 experimental measurements, where 85% of these samples were used to train the NN and the remaining 15% from the same environment was used to test the NN accuracy. In our approach, we subdivided the available samples in a similar way, using 80% of the data to train the NN and 20% of the data to preliminarily assess the NN accuracy. In addition, we also extensively assess the NN accuracy on a different layout, not used during training. In Zineb and Ayadi [2016], the input variables were the transmitter‐receiver distance, the frequency, the wall attenuation, and the floor attenuation. All input variables were measured as absolute coordinates, whereas in our approach input variables were expressed in coordinates relative to the observation point in the building where the NN has to predict the exposure field. The approach in Zineb and Ayadi [2016] modeled only the field exposure generated by APs at fixed positions inside the building. It could predict the path loss with a mean error of 0.7 dB and SD of 5.22 dB; this was the best performance the authors achieved with an NN of three hidden layers. In our study, we found that the best performance of the NN was achieved with a similar number of layers, that is, with four to five hidden layers. Our NN could predict the true electric field was of the same accuracy as in Zineb and Ayadi [2016] (median *Δdb*: −0.4 to 0.6 dB, *RMS*_*Δdb*_; 2.5–5.1 dB). However, our approach is more generalized as it could successfully estimate the electric field in a more complex scenario than in Zineb and Ayadi [2016], comprising the contributions due to WiFi users and clients in addition to the contribution of the transmitting APs.

## CONCLUSIONS

We investigated the feasibility and accuracy of an NN approach to model and estimate the field of exposure generated by WiFi sources in indoor scenarios. Our approach could model the electric field generated in propagation conditions more complex than those typically addressed in past ML studies as it considered the effects of multiple sources during both DL and UL transmissions. It was shown that the proposed method also performs well for different environments and is thus generally usable. As a matter of fact, the proposed NN was able to predict the electric field with a median error equal to −0.4 to 0.6 dB and an RMS error of 2.5–5.1 dB in the layout used during training and in a different layout. The accuracy of the proposed NN model was comparable or in some cases even better than that of previous approaches based on ML that addressed only DL exposure. Future works will consider how to further improve the flexibility of the proposed NN to address more generalized scenarios to include the effects of 4 G and 5 G devices.

## Supporting information

Supporting information.
