# Supplementary material for: Use of Machine Learning for the Estimation of Down‐ and Up‐Link Field Exposure in Multi‐Source Indoor WiFi Scenarios
Source: Bioelectromagnetics. 2021 Jul 23;42(7):550–61. doi: 10.1002/bem.22361 (PMC8519090; doi:10.1002/bem.22361)
Supplement: Supplementary file 1 — Supporting information. [file BEM-42-550-s001.docx]

***APPENDIX***

**Table 1supplementary – Values of *KMSE* (x 10^-4^) as a function of the partition and the number of layers for the NN model built on DL data.**

| Partition # | Number of layers | | | | | | | | | |
| --- | --- | --- | --- | --- | --- | --- | --- | --- | --- | --- |
|  | 1 | 2 | 3 | 4 | 5 | 6 | 7 | 8 | 9 | 10 |
| 1 | 14 | 13.9 | 13.5 | 13.1 | 6.5 | 7.6 | 7.2 | 7.3 | 8.2 | 9.6 |
| 2 | 5.6 | 7.3 | 4.9 | 5.1 | 4.5 | 4.9 | 5.3 | 5.3 | 5.1 | 5 |
| 3 | 23.8 | 14.4 | 14.9 | 13.2 | 15.7 | 12.7 | 14 | 12.2 | 14.4 | 12.9 |
| 4 | 25 | 27.2 | 24.2 | 16.9 | 14.3 | 17.1 | 14.6 | 15.2 | 14.9 | 16.2 |
| 5 | 37.8 | 41.8 | 30.4 | 25.4 | 23.7 | 29.5 | 17.5 | 20.2 | 20.9 | 22 |
| 6 | 105.7 | 22.3 | 24.6 | 24.3 | 18.2 | 22.7 | 19.5 | 17.7 | 18.7 | 16.8 |
| 7 | 6.3 | 5 | 4.4 | 4.7 | 5 | 4.1 | 3.7 | 4.2 | 4.3 | 4.2 |
| 8 | 8.9 | 6.4 | 6.4 | 6.3 | 5.8 | 5.9 | 6 | 5.8 | 6 | 5.7 |
| 9 | 33 | 14.2 | 24.7 | 17.9 | 18.5 | 17 | 17.1 | 15.3 | 19.7 | 17 |
| 10 | 35.5 | 20.9 | 12.7 | 14.8 | 14.4 | 15 | 20.7 | 19.5 | 19.1 | 18.7 |

**Table 2supplementary – Values of *KMSE* (x 10^-4^) as a function of the partition and the number of layers for the NN model built on DL+UL|clients data.**

| Partition # | Number of layers | | | | | | | | | |
| --- | --- | --- | --- | --- | --- | --- | --- | --- | --- | --- |
|  | 1 | 2 | 3 | 4 | 5 | 6 | 7 | 8 | 9 | 10 |
| 1 | 23.3 | 22.1 | 22.1 | 22.4 | 22.8 | 22.5 | 22.3 | 22 | 23 | 23.3 |
| 2 | 73.7 | 27 | 18.6 | 13.7 | 12.7 | 13 | 13.8 | 14.2 | 13.8 | 13.1 |
| 3 | 46.3 | 40.7 | 45.4 | 42.2 | 40.3 | 34.6 | 40.5 | 40.4 | 40.4 | 40.6 |
| 4 | 57.8 | 68.3 | 27.1 | 24.7 | 29.7 | 27.6 | 26 | 26.6 | 27.2 | 22.2 |
| 5 | 16.8 | 15.7 | 17.6 | 18 | 18.2 | 16.3 | 17.5 | 16 | 17.8 | 15.4 |
| 6 | 106.7 | 35.7 | 31.3 | 29.2 | 28.7 | 31.6 | 29.7 | 30.2 | 30.7 | 30.5 |
| 7 | 29 | 23.4 | 24.4 | 24 | 26.7 | 25.9 | 23 | 24.4 | 24.9 | 25.6 |
| 8 | 33.3 | 40.9 | 41.7 | 39.9 | 45.6 | 44.2 | 34.4 | 36.7 | 37.9 | 35.3 |
| 9 | 44 | 43.5 | 49.3 | 40.6 | 45.3 | 47.8 | 42.8 | 42.3 | 44.8 | 43.4 |
| 10 | 31.1 | 17.4 | 14.9 | 14.7 | 18.1 | 18.6 | 19.4 | 16.3 | 17.9 | 17.9 |

**Table 3supplementary – Values of *KMSE* (x 10^-4^) as a function of the partition and the number of layers for the NN model built on DL+UL|clients&users data.**

| Partition # | Number of layers | | | | | | | | | |
| --- | --- | --- | --- | --- | --- | --- | --- | --- | --- | --- |
|  | 1 | 2 | 3 | 4 | 5 | 6 | 7 | 8 | 9 | 10 |
| 1 | 35 | 50.2 | 35.5 | 32 | 32 | 33.1 | 31.8 | 31.2 | 33.1 | 34.2 |
| 2 | 53.8 | 56.8 | 45.4 | 48.4 | 42.7 | 52.2 | 44.5 | 43.7 | 44.9 | 44.6 |
| 3 | 53.7 | 67.5 | 62.1 | 52.8 | 48.8 | 49.8 | 53.9 | 51.9 | 55.1 | 52.8 |
| 4 | 101.8 | 47.2 | 41.2 | 42.6 | 40.3 | 40.8 | 42.4 | 40.5 | 39.6 | 40.9 |
| 5 | 133 | 39.2 | 36.8 | 35 | 34.2 | 35.1 | 36.2 | 34.6 | 34.6 | 35.4 |
| 6 | 43.6 | 64.5 | 39.6 | 41.2 | 39.2 | 38.3 | 40.5 | 40.8 | 37.6 | 32.9 |
| 7 | 61.3 | 63.2 | 64.2 | 61.3 | 58 | 54.2 | 59.3 | 59.5 | 59.3 | 60.7 |
| 8 | 46.7 | 104.9 | 45 | 46.9 | 46 | 46 | 45.4 | 47 | 47.2 | 46.3 |
| 9 | 33.5 | 33 | 33.9 | 31.8 | 33.5 | 32.7 | 32.7 | 33.4 | 32.2 | 32.6 |
| 10 | 53.4 | 50.6 | 46.6 | 44.1 | 44.6 | 47.2 | 45.6 | 51.9 | 45.3 | 49.8 |

**Figure 1supplementary – Cross-validation score *CVS* as a function of the number of layers for the NN model built on DL, DL+UL|clients, and DL+UL|clients&users data. *CVS* was calculated by eq. (3) using the *KMSE* values reported in the tables above.**


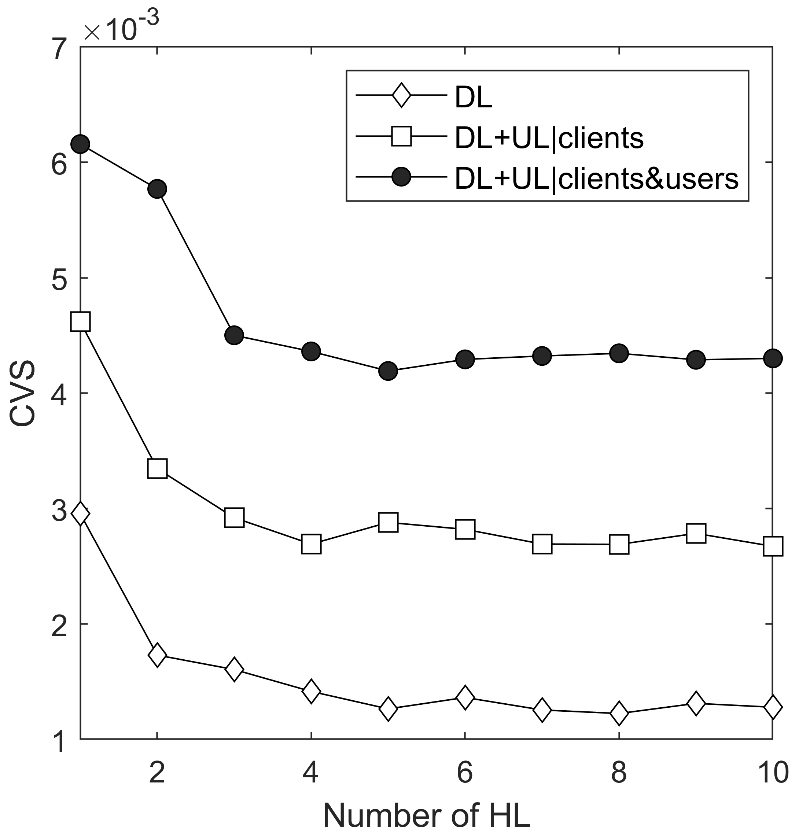


**Table 4supplementary – The format of the input vector *x* to the NN model built on DL, UL+UL|clients, and DL+UL|clients&users data(*).**

| NN model | Input vector *x* |
| --- | --- |
| DL | $x=[{\#AP}_{\leq50};{\#AP}_{50-100};\cdots;{\#AP}_{450-500};{\#WA}_{\leq50};\cdots;{\#WA}_{450-500};$  ${\#MWA}_{\leq50};\cdots;{\#MWA}_{450-500};{\#PL}_{\leq50};\cdots;{\#PL}_{450-500}]$ |
| UL+UL\|clients | $x=[{\#AP}_{\leq50};{\#AP}_{50-100};\cdots;{\#AP}_{450-500};{\#WA}_{\leq50};\cdots;{\#WA}_{450-500};$  ${\#MWA}_{\leq50};\cdots;{\#MWA}_{450-500};{\#PL}_{\leq50};\cdots;{\#PL}_{450-500};$  ${\#CL}_{\leq50};\cdots;{\#CL}_{450-500}]$ |
| DL+UL\|clients&users | $x=[{\#AP}_{\leq50};{\#AP}_{50-100};\cdots;{\#AP}_{450-500};{\#WA}_{\leq50};\cdots;{\#WA}_{450-500};$  ${\#MWA}_{\leq50};\cdots;{\#MWA}_{450-500};{\#PL}_{\leq50};\cdots;{\#PL}_{450-500};$  ${\#CL}_{\leq50};\cdots;{\#CL}_{450-500};{\#US}_{\leq50};\cdots;{\#US}_{450-500}]$ |

${\#AP}_{\leq50};{\#AP}_{50-100};\cdots;{\#AP}_{450-500}:$number of APs at distances from within 50 cm up to 5 m from the point in the room where the E field has to be calculated.

${\#WA}_{\leq50};\cdots;{\#WA}_{450-500}$: number of non-metallic walls at distances from within 50 cm up to 5 m from the point in the room where the E field has to be calculated.

${\#MWA}_{\leq50};\cdots;{\#MWA}_{450-500}$: number of metallic walls at distances from within 50 cm up to 5 m from the point in the room where the E field has to be calculated.

${\#PL}_{\leq50};\cdots;{\#PL}_{450-500}$: average path loss of non-metallic walls at distances from within 50 cm up to 5 m from the point in the room where the E field has to be calculated.

${\#CL}_{\leq50};\cdots;{\#CL}_{450-500}$: number of WIFI clients at distances from within 50 cm up to 5 m from the point in the room where the E field has to be calculated.

${\#US}_{\leq50};\cdots;{\#US}_{450-500}$: number of WIFI users at distances from within 50 cm up to 5 m from the point in the room where the E field has to be calculated.

(*) the multi-dimensional Matlab ‘network structure’ file containing the cell arrays with the weight and connections among the layers and all the other information related to the structure of each NN model is available upon request to the Corresponding Author.
